# Relationship between Pb ion off-centering and lone pair electrons

**DOI:** 10.1038/s41598-025-93984-5

**Published:** 2025-03-18

**Authors:** Kosuke Kurushima, Hiroshi Nakajima, Takahiro Ogata, Yuki Sakai, Masaki Azuma, Shigeo Mori

**Affiliations:** 1 Toray Research Center, Otsu, Shiga 520-8567 Japan; 2 Department of Materials Science, Osaka Metropolitan University, Sakai, Osaka 599-8531 Japan; 3 Materials and Structures Laboratory, Institute of Integrated Research, Institute of Science Tokyo, 4259 Nagatsutacho, Midoriku, Yokohama 226-8501 Japan; 4 Kanagawa Institute of Industrial Science and Technology (KISTEC), Shimoimaizumi, Ebina, Kanagawa 243-0435 Japan; 5 Research Center for Autonomous System Materialogy, Institute of Integrated Research, Institute of Science Tokyo, 4259 Nagatsutacho, Midoriku, Yokohama 226-8501 Japan

**Keywords:** Electronic properties and materials, Ferroelectrics and multiferroics, Imaging techniques, Characterization and analytical techniques

## Abstract

High-pressure synthesized Pb-based perovskites exhibit diverse functional properties. PbCrO_3_, for instance, displays distinctive diffuse scattering and valence skipping, forming Pb^2+^ and Pb^4+^ ions. However, the spatial distribution of Pb ions in the crystal remains largely unexplored. Here, we elucidate the role of Pb ions with different valences through Sr substitution, using high-resolution transmission electron microscopy combined with elemental mapping. This approach allows us to accurately examine the distribution of Sr and Pb ions in the same atomic columns. The results reveal that in Pb_0.8_Sr_0.2_CrO_3_, Sr ions occupy a squared lattice, while Pb ions exhibit positional distortions. Simulations based on the experimentally determined Pb distribution reproduce the diffuse scattering observed in PbCrO_3_. These findings suggest that the lone pair electrons of *s* orbitals are responsible for the local lattice distortion. Our study provides atomistic insights into the local structures of materials exhibiting valence skipping.

## Introduction

Lead is a widely used metallic element known for its high workability and relatively simple refining process^1^. It remains essential in various applications, including crystal glass, pigments, soundproofing materials, and electronic components. Despite its toxicity and the urgent need for safer alternatives^2^, Pb’s high functional value makes replacement challenging, particularly in critical applications such as storage batteries and X-ray shielding. Additionally, perovskite Pb(Zr, Ti)O_3_ is a key Pb-based functional material^3–7^ with exceptional piezoelectric properties, for which no viable substitutes currently exist. Notably, it is exempt from the RoHS Directive (EU directive on the restriction of hazardous substances in electronic and electrical equipment). Understanding the mechanisms behind the unique properties of Pb compounds is crucial for developing effective alternatives and advancing toward a Pb-free society.

Pb-containing perovskite-type oxides exhibit various functional properties beyond ferroelectricity and piezoelectricity, including applications in solar cells, catalysis, and metal–insulator transitions^8,9^. The physical properties of perovskite oxides are often primarily attributed to the *B*-site ion in the *AB*O_3_ structure. However, when Pb occupies the *A*-site, its characteristics can play a crucial role in determining the crystal structure and overall properties. As a heavy element, Pb possesses 6*s* orbital electrons, which contribute to the unique physical properties of Pb-based perovskites. The electron configuration of Pb is [Xe]4*f*^14^5*d*^10^6*s*^2^6*p*^2^, while Pb^2+^ is [Xe]4*f*^14^5*d*^10^6*s*^2^, and Pb^4+^ is [Xe]4*f*^14^5*d*^10^6*s*^0^. Hereafter, the electron configuration of Pb^2+^ is denoted as 6*s*^2^ and that of Pb^4+^ as 6*s*^0^. In Pb^2+^-containing crystals, the lone-pair electrons of 6*s*^2^ are believed to induce structural distortions that break inversion symmetry. Experimental studies have confirmed anisotropic covalent bonding between Pb and O ions in PbTiO_3_^10^. Although some PbTiO_3_ derivatives can be synthesized at ambient pressure^11^, many perovskite oxides with distinctive properties, composed of Pb and 3*d* transition metals, have recently been reported using high-pressure synthesis methods^12–16^. In certain materials, Pb exhibits valence flexibility owing to the presence or absence of electrons in the 6*s* orbital, a phenomenon known as valence skipping^17,18^. Specifically, Pb^2+^ (6*s*^2^) and Pb^4+^ (6*s*^0^) states are stabilized, whereas the intermediate Pb^3+^ (6*s*^1^) configuration is inherently unstable.

Perovskite PbCrO_3_ is an interesting material with a unique structure and physical properties attributed to valence skipping. Recently, a “charge glass” state of Pb^2+^_0.5_Pb^4+^_0.5_Cr^3+^O_3_ was reported in PbCrO_3_^19^. The study also revealed that under pressure, charge transfer occurs between Pb^4+^ and Cr^3+^, leading to the formation of Pb^2+^Cr^4+^O_3_. This charge redistribution results in a considerable change in the Cr–O bonding distance, causing a volume shrinkage of up to 10%, which is promising for developing giant negative thermal expansion materials based on PbCrO_3_. Additionally, its local structure exhibits distinctive features because high-angle annular dark-field scanning transmission electron microscopy (HAADF–STEM) images have shown an off-center displacement of Pb ions^19,20^. The observations revealed regions where Pb and Cr ions retained an undistorted cubic lattice, indicating that not all Pb ions were displaced^20^. This suggests that the presence or absence of off-centering may be influenced by the valence states of Pb^2+^ and Pb^4+^ in the charge glass state. R. Yu et al. constructed a structural model of diffuse scattering caused by Pb displacements, proposing a threefold structure based on pair-distribution function analysis^19^. However, discrepancies remain between experimental and simulation data because the diffuse scattering is not strictly confined to the threefold position but is instead located between the threefold and fourfold positions^20^. These unusual phenomena may stem from the valence flexibility of Pb ions, i.e., valence skipping. Furthermore, it is unclear whether Pb^2+^ and/or Pb^4+^ are responsible for off-centering. Therefore, further investigation is needed to clarify the origin of the characteristic diffuse scattering observed in PbCrO_3_.

In this study, we investigate Sr-substituted PbCrO_3_ to gain insight into diffuse scattering. When the solid solution Pb_1–*x*_Sr_*x*_CrO_3_ is formed at *x* = 0.2, the coexistence of a Pb^2+^_0.5_Pb^4+^_0.5_Cr^3+^O_3_-like phase and a Pb^2+^Cr^4+^O_3_-like phase has been reported^19^. To maintain charge balance, the valence state of the former phase is considered to be Pb^2+^_0.3_Sr^2+^_0.2_Pb^4+^_0.5_Cr^3+^O_3_. Because Sr^2+^ ions substitute at Pb sites, they are expected to reduce the number of Pb^2+^ ions. Moreover, the role of Pb^4+^ in PbCrO_3_ can be inferred through Sr^2+^ substitution because Sr^2+^ lacks lone-pair electrons in its *s* orbitals. An additional advantage of Sr substitution is that it allows atomic displacements of both Pb and Sr ions to be observed in the same atomic columns using transmission electron microscopy. We investigated the local structure of Pb_0.8_Sr_0.2_CrO_3_, synthesized under high pressure, to clarify the origin of diffuse scattering. Using high-resolution scanning transmission electron microscopy (STEM) and atomic-resolution energy-dispersive X-ray spectroscopy (EDS), we analyzed structural details, including Pb off-centering. Our findings reveal that Pb off-centering is strongly related to the inactive lone-pair electrons of Pb^2+^. Furthermore, this study proposes a method for estimating the distribution of Pb^2+^ and Pb^4+^ ions in the charge glass state through Sr^2+^ substitution using atomic-resolution EDS mapping.

## Results and discussion

**Fig. 1:**
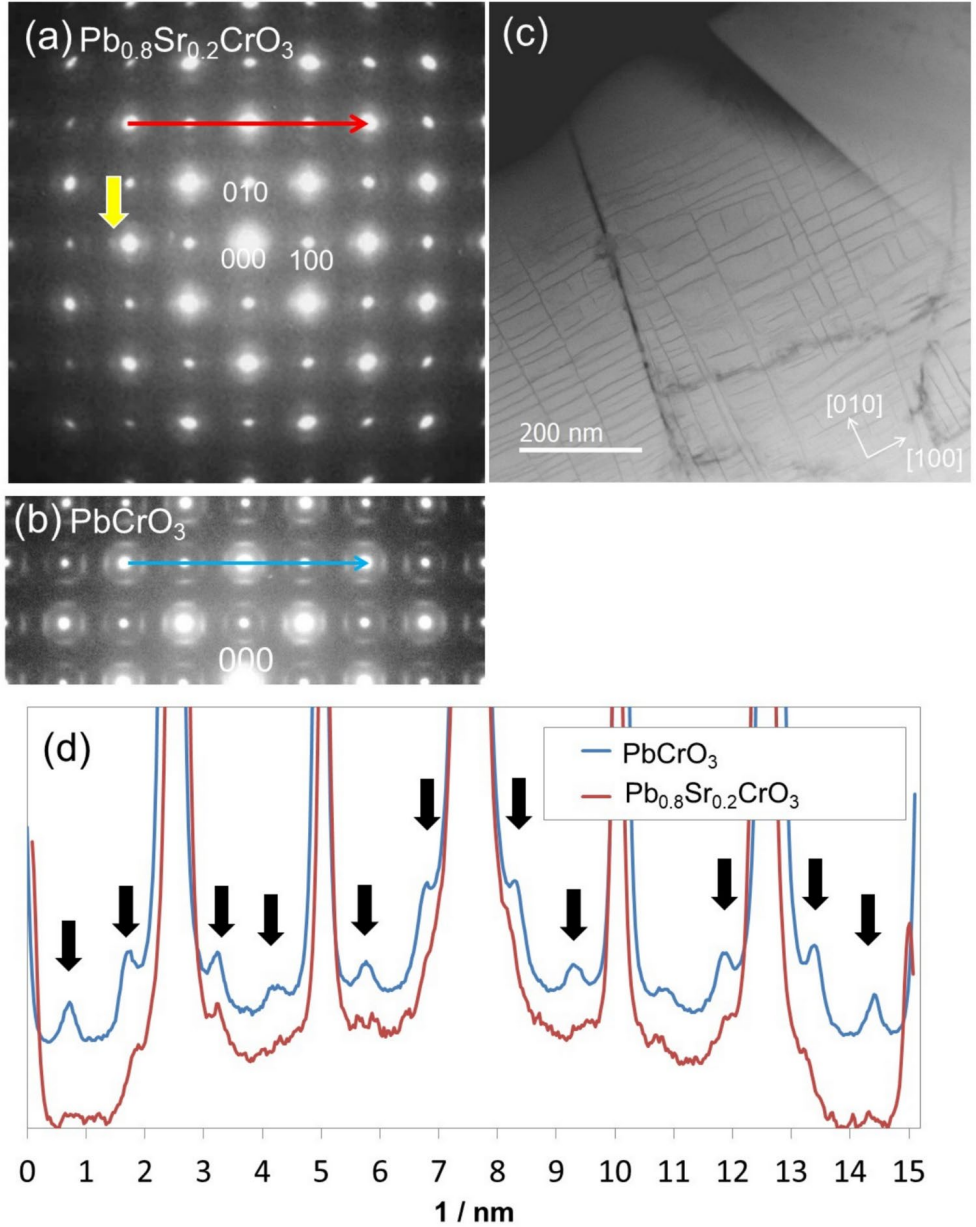
Observation of diffuse scattering in Pb_0.8_Sr_0.2_CrO_3_. (**a**) Selected-area electron diffraction pattern of Pb_0.8_Sr_0.2_CrO_3_. (**b**) Selected-area electron diffraction pattern of PbCrO_3_ for comparison. (**c**) Low-magnification HAADF–STEM image of the (001) plane of Pb_0.8_Sr_0.2_CrO_3_. (**d**) Intensity profile of the diffuse scattering at the arrow positions in panels (**a**) and (**b**).

Figure 1 shows the observation results of Pb_0.8_Sr_0.2_CrO_3_ along the [00$\overline{1 }$] axis. Figure 1a shows the electron diffraction pattern, which exhibits characteristic diffuse scattering (e.g., yellow arrow in the figure), suggesting that the structural features of PbCrO_3_ (Fig. 1b) are preserved in the matrix phase. The low-magnification HAADF–STEM image in Fig. 1c reveals straight contrast in the plane, indicating a mixed crystalline and amorphous microstructure. Previous studies on pure PbCrO_3_ have shown a combination of crystalline and amorphous regions in a single crystal grain^20^. This feature also remains consistent in Sr-substituted PbCrO_3_. Figure 1d shows the intensity profiles at the positions indicated by the red and blue arrows in Fig. 1a, b. When comparing PbCrO_3_ with Pb_0.8_Sr_0.2_CrO_3_, the diffuse scattering intensity in Pb_0.8_Sr_0.2_CrO_3_ is reduced at the locations marked by the black arrows. This decrease in intensity suggests that the reduction is linked to a decrease in the number of Pb^2+^ ions with inactive lone pair electrons resulting from Sr^2+^ substitution: Pb^2+^ ions may be responsible for the Pb off-center behavior.

**Fig. 2:**
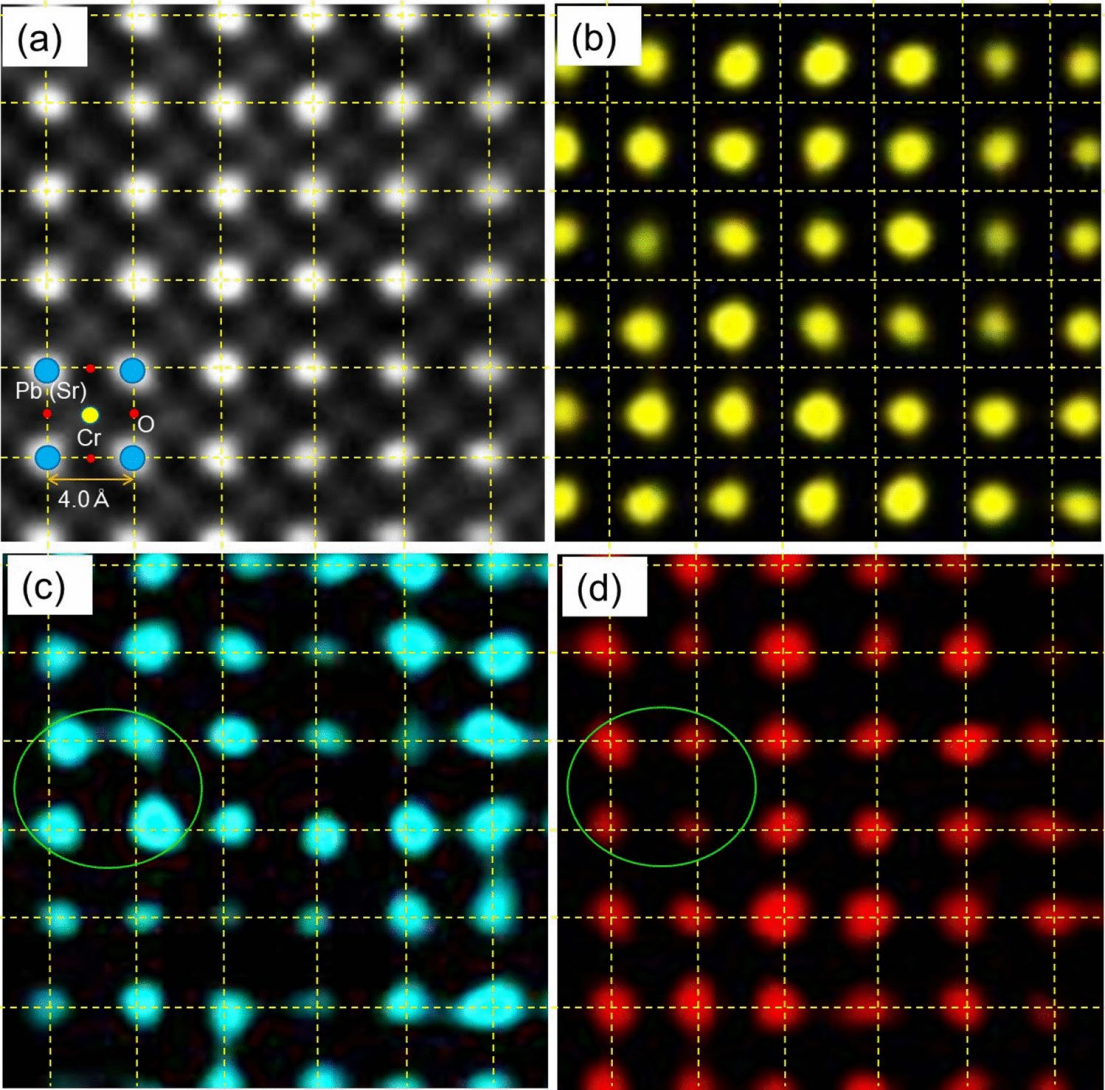
Atomic-scale configurations of each atom in Pb_0.8_Sr_0.2_CrO_3_. (**a**) HAADF–STEM image. Energy dispersive X-ray spectroscopy images using (**b**) Cr *K*, (**c**) Pb *L*, and (**d**) Sr *L* absorption peaks. The yellow grid lines are added as eye guides to confirm atomic displacements.

Atomic-resolution EDS observations were performed on Pb_0.8_Sr_0.2_CrO_3_, with the mapping results for each element presented in Fig. 2. Figure 2a shows a HAADF image where the bright spots correspond to the Pb positions at the *A* site. Figure 2b, c confirms that Cr occupies the *B* site and Pb the *A* site, as indicated by the crystal structure model (inset in Fig. 2a). When comparing Fig. 2c, d, it is evident that Sr is incorporated into the *A* site. It is important to note that the high and low intensities correspond to the number of atoms present in each column: columns with high Pb intensity exhibit low Sr intensity. These elemental maps reveal that Sr and Pb occupy different positions in the same *A* site, even though they were acquired simultaneously.

The dotted grid lines in the figure indicate that Sr is typically located at the intersection points. In contrast, the bright spots in the Pb map appear distorted or slightly displaced from the grid. Sr^2+^ (5*s*^0^) ions are found to occupy the lattice points, suggesting that Pb^4+^ (6*s*^0^) ions remain undisturbed owing to their similar electronic configurations, which lack lone pair electrons. Because Pb ions can exist in either divalent or tetravalent states and Pb^4+^ ions are assumed to be at the lattice points, the Pb ions slightly off-center from these lattice points are likely Pb^2+^ ions. Pb ions with a 6*s*^2^ configuration, possessing lone pair electrons, would distort the coordination environment in the crystal, causing off-centering similar to what is observed in ferroelectric perovskites.

**Fig. 3:**
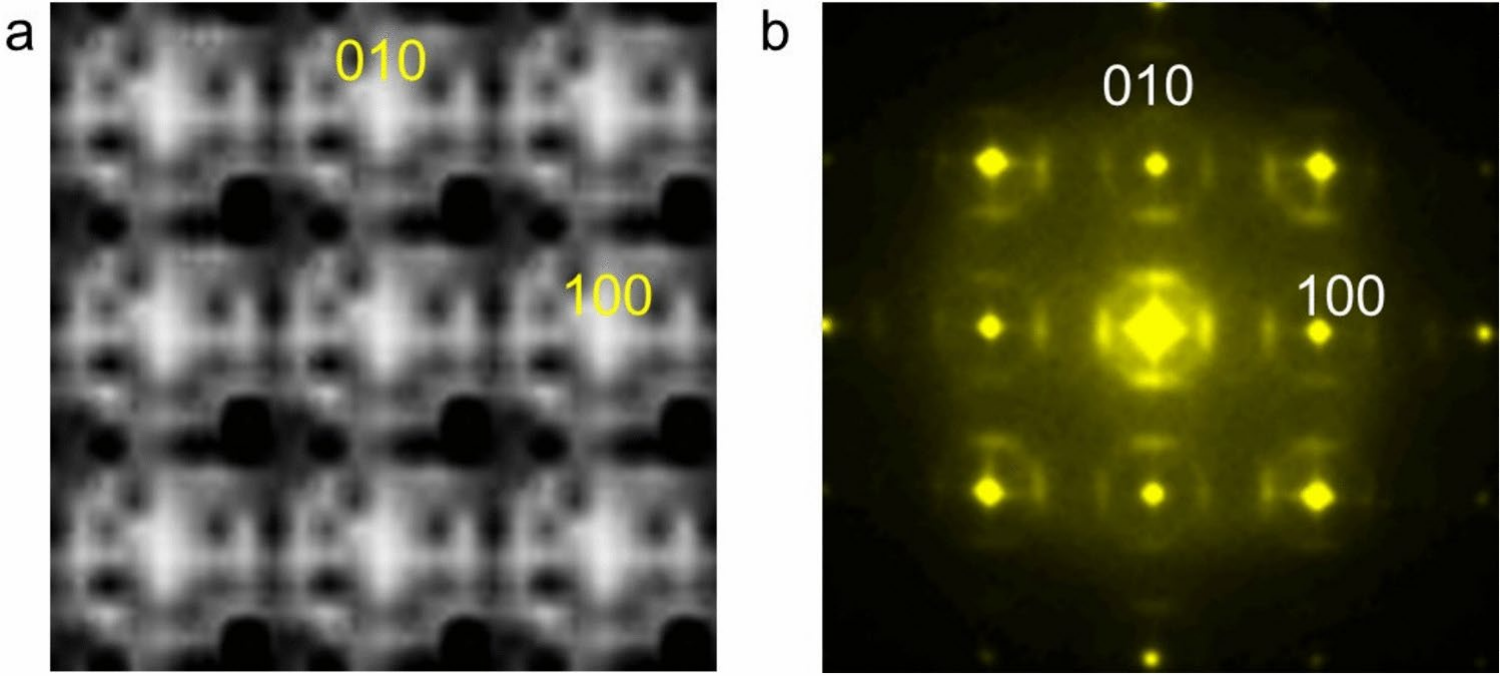
Diffuse scattering analysis. (**a**) Simulated pattern calculated using Pb atom coordinates derived from observations. (**b**) Fast Fourier transform (FFT) pattern from a high-resolution TEM image.

To better understand the origin of the diffuse scattering, we simulated a diffraction pattern based on the positions of the Pb atoms. Figure 3a shows the diffuse scattering simulation using a model structure derived from the coordinates extracted from the Pb *L* map. The simulation was performed with a small number of 42 Pb atoms (2.5 nm^2^ range). Nonetheless, the simulation results qualitatively reproduce the characteristics of the diffuse scattering, which is consistent with the fast Fourier transform (FFT) pattern of a high-resolution TEM image shown in Fig. 3b. This confirms that the displacement of Pb atoms is responsible for this characteristic diffuse scattering. Notably, the simulated pattern shows intensity maxima at the threefold positions, while the experimental FFT pattern displays diffuse maxima. The charge glass state may blur the peaks of the diffuse scattering. The local off-centering of Pb^2+^ ions is attributed to the lone-pair effect of the 6*s*^2^ electrons, resulting from *s*–*p* orbital hybridization, as observed in other materials^10,21–23^. This hybridization interaction can be influenced by coordination. As a result, the charge glass state may locally change the amplitude of off-centering (i.e., the distance from the centrosymmetric position for each ion), as shown in Fig. 2c. This variation in off-centering amplitude could cause the threefold peak to disperse, as seen in the experimental diffraction pattern. This accounts for the earlier observation that the diffuse scattering occurs between the threefold and fourfold positions^20^. As a direction for future work, spectroscopic techniques such as extended X-ray absorption fine structure (EXAFS) could provide deeper insights into the local structure surrounding individual ions by selecting Pb, Cr, and Sr X-ray absorption edges. EXAFS is capable of detecting atomic displacements in the unit cell, offering information that could validate the microscopy images presented in this study.

**Fig. 4:**
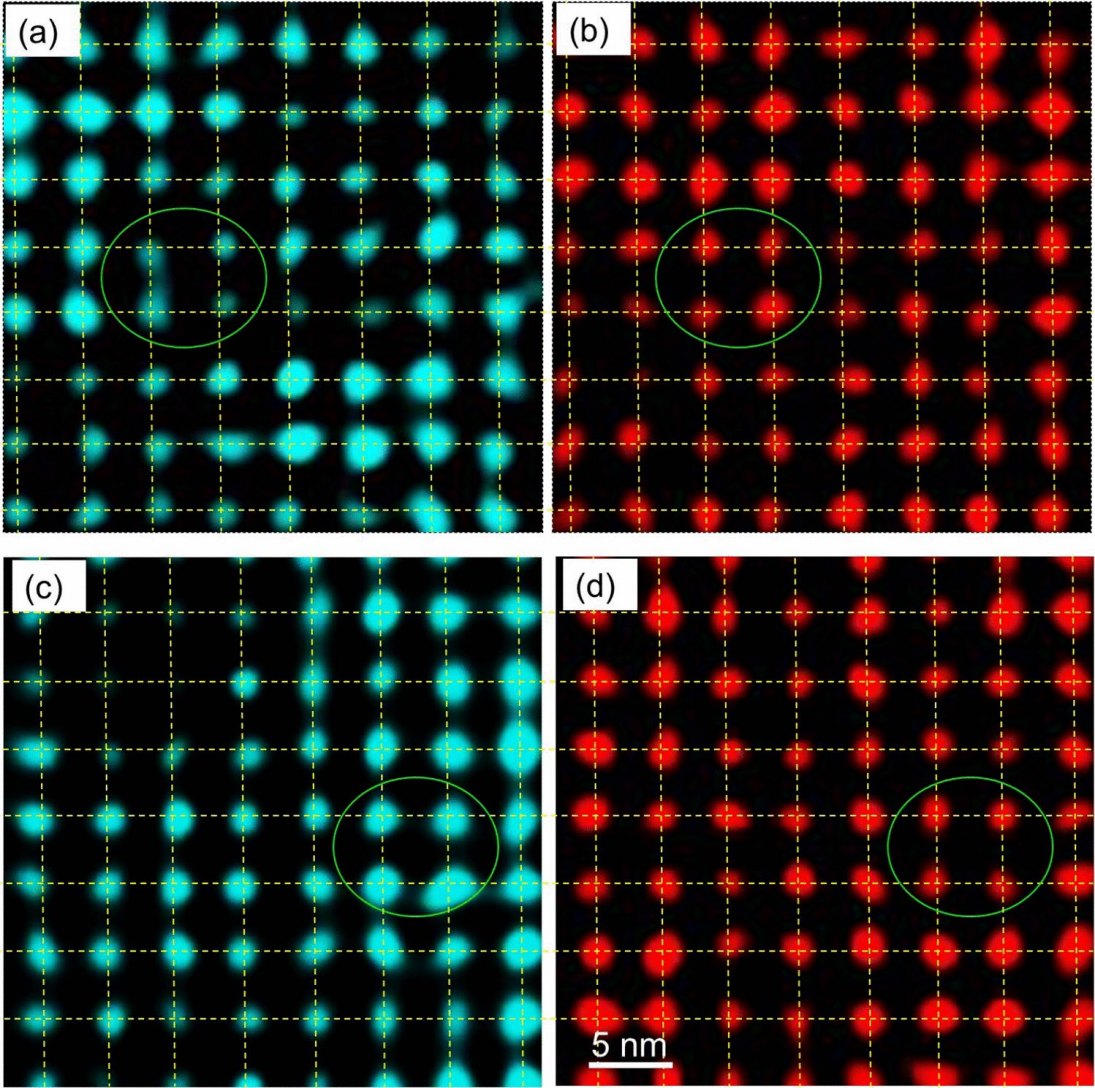
Observation of atomic-scale configuration in other regions. (**a**, **b**) Pb and Sr ion maps in the same field of view. (**c**, **d**) Observation of a different area in the same grain. These results were obtained from a grain different from that in Fig. 2. The energies used are (**a**, **c**) Pb *L* and (**b**, **d**) Sr *L* absorption peaks.

We show that Pb ions undergo local displacement throughout the entire region of a grain. Figure 4 shows high-resolution EDS maps of the (001) planes of Pb_0.8_Sr_0.2_CrO_3_ from a grain different from that in Fig. 2. These maps are obtained using Pb *L* and Sr* L* absorption peaks. Square grid lines are included to aid visual interpretation. The green circles in Fig. 4a, c highlight regions where Pb ions are displaced from their lattice positions. These circles clearly indicate that the Pb ions are off the lattice. Thus, the results from Fig. 2 are confirmed in other areas. It is important to note that the grains shown in Figs. 2 and 4 likely exhibit the (Pb^2+^, Pb^4+^)Cr^3+^O_3_ phase because this phase induces local displacements of Pb ions, while the other phase, Pb^2+^Cr^4+^O_3_, is an ordered state without distortion. X-ray diffraction studies^19^ indicate that these two phases are mixed in the synthesized specimen. This observation confirmed that the two phases existed in different grains, with the grains of (Pb^2+^, Pb^4+^)Cr^3+^O_3_ exhibiting atomic displacement throughout the entire grain. Additionally, Fig. 4 shows a substantial displacement along the <100> direction, which corresponds to the high diffuse scattering intensity observed in this direction. Relatively weaker displacements are seen along the oblique < 110> direction, consistent with the finding that the intensity distribution of the diffuse scattering is nearly spherical^20^. These results further support the conclusion that this distortion pattern is the origin of the characteristic diffuse scattering in PbCrO_3_.

## Summary

STEM analysis and atomic-resolution EDS mapping were used to examine the microstructural features and identify the origin of the observed diffuse scattering in the charge glass PbCrO_3_ upon partial substitution of Sr ions. The Pb_0.8_Sr_0.2_CrO_3_ specimen exhibits a phase coexistence of crystalline and amorphous regions, along with diffuse scattering around the fundamental reflections in the electron diffraction pattern, similar to that of PbCrO_3_. This indicates that Sr substitution does not disrupt the intrinsic composite structure and charge glass state of PbCrO_3_. The distribution of Pb^2+^ and Pb^4+^ can be inferred from the atomic-resolution EDS maps, which reveal Sr^2+^ positions at on-centered sites. Because Pb^4+^ lacks lone pair electrons, similar to Sr^2+^, Pb^4+^ ions are expected to occupy on-centered positions in PbCrO_3_. Consequently, Pb^2+^ ions, which possess lone pair electrons, are off-centered, contributing to the characteristic diffuse scattering. These behaviors resemble those of ferroelectric materials, such as relaxors, which exhibit small local distortions owing to the inactive lone pair electrons of Pb^2+^ ions^24–27^. We believe these atomistic insights could be valuable for designing Pb-free functional materials with high performance.

As a remark, elements such as Tl, Pb, Bi, Sn, and Sb cause valence skipping^17^, allowing them the flexibility to adopt either the *s*^2^ or *s*^0^ electron configuration in their 5*s* and 6*s* orbitals. Off-centering is frequently observed in materials containing these elements. Studies based on density functional calculations indicate that lone pair electrons contribute to the distortion^28^. However, establishing a structure, particularly for a randomly displaced configuration, has proven difficult because X-ray diffraction only provides information on average structures. The observation methods presented in this study will be useful for investigating local structures in materials exhibiting valence skipping.

## Methods

High-pressure synthesis is an effective method for stabilizing the high-density structure of *AB*O_3_ (where *A* and *B* represent cations). The Pb_0.8_Sr_0.2_CrO_3_ polycrystalline specimen was synthesized following the same procedure as PbCrO_3_^19,20^, using a cubic-anvil high-pressure synthesis technique. A powder mixture is enclosed in a platinum capsule, placed in a pyrophyllite cell, then pressurized and heated using a graphite heater.

Observations were performed using a JEM-ARM200F with aberration correctors (JEOL Co. Ltd.). The instrument is equipped with two 100 mm^2^ silicon-drift detectors, enabling atomic-resolution EDS observations^29,30^. The accelerating voltage was set to 200 kV, and all measurements were performed at room temperature. Because elemental maps were filtered to reduce noise, discussing absolute quantities based on intensity was challenging. Local structural analysis using STEM is particularly useful for specimens prepared by high-pressure synthesis owing to the ability to observe a small amount of sample and to distinguish grains of target materials from those of impurities.

For TEM thin foils, the powder specimen is embedded in epoxy resin, placed between Si wafers, and cut into approximately 3 mm^2^ pieces. After mechanical polishing to a thickness of approximately 10 μm, it was attached to a molybdenum single-hole mesh. The mesh is further processed by Ar-ion milling while rotating until a hole is formed. The cross section at the edge of the perforated sample has a wedge shape, and the area near the hole represents the thin film region that can be observed by TEM. This milling method offers the advantage of preserving the original structure without the damage typically caused by thin-film processing with focused ion beams. Additionally, the Ar-ion milling method allows for a 100 µm field-of-view, making it easier to search grains with target crystal orientations.

An electron diffraction pattern was simulated using xHREM (HREM Research Inc.). A multislice method based on a supercell was used to calculate the diffuse scattering intensity. The supercell for the simulation was constructed from EDS elemental maps. Ionic positions were determined from Sr and Pb elemental maps by automatically measuring the center position of each ion. Sr ion sites were assumed to be at the origin without distortion, and the displacement of Pb sites was calculated as the distance from the origin. In total, 42 Pb sites were extracted from the EDS maps.

## Data Availability

The data that support the findings of this study are available from the corresponding author upon reasonable request.
